# Supplementary material for: Resolving the full spectrum of human genome variation using Linked-Reads
Source: Genome Res. 2019 Apr;29(4):635–45. doi: 10.1101/gr.234443.118 (PMC6442396; doi:10.1101/gr.234443.118)
Supplement: Supplemental Material [file supp_gr.234443.118_Supplemental_longranger-2.2.zip › longranger-2.2.tar.bz2!/lr2.2-public/lib/stan/cmdstan/stan/doc/api/html/math_2functions_2bessel__second__kind_8hpp_source.html]

Stan: src/stan/math/functions/bessel\_second\_kind.hpp Source File


|  |  |
| --- | --- |
|  | Stan  2.5.0  probability, sampling & optimization |


- Main Page
- Related Pages
- Namespaces
- Classes
- Files

- File List
- File Members

All Classes Namespaces Files Functions Variables Typedefs Enumerations Enumerator Friends Macros Pages

- src
- stan
- math
- functions

bessel\_second\_kind.hpp

Go to the documentation of this file.

1 #ifndef STAN\_\_MATH\_\_FUNCTIONS\_\_BESSEL\_SECOND\_KIND\_HPP

2 #define STAN\_\_MATH\_\_FUNCTIONS\_\_BESSEL\_SECOND\_KIND\_HPP

3

4 #include <boost/math/special\_functions/bessel.hpp>

5

6 namespace stan {

7  namespace math {

8

38  template <typename T2>

39  inline T2

40  bessel\_second\_kind(const int v, const T2 z) {

41  return boost::math::cyl\_neumann(v,z);

42  }

43

44  }

45 }

46

47 #endif

stan::math::bessel\_second\_kind

T2 bessel\_second\_kind(const int v, const T2 z)

**Definition:** bessel\_second\_kind.hpp:40


---

[ Stan Home Page ]

*© 2011–2014,
Stan Development Team.*
